# The White-Nose Syndrome Transcriptome: Activation of Anti-fungal Host Responses in Wing Tissue of Hibernating Little Brown Myotis

**DOI:** 10.1371/journal.ppat.1005168

**Published:** 2015-10-01

**Authors:** Kenneth A. Field, Joseph S. Johnson, Thomas M. Lilley, Sophia M. Reeder, Elizabeth J. Rogers, Melissa J. Behr, DeeAnn M. Reeder

**Affiliations:** 1 Department of Biology, Bucknell University, Lewisburg, Pennsylvania, United States of America; 2 Department of Pathobiological Sciences, School of Veterinary Medicine, University of Wisconsin-Madison, Madison, Wisconsin, United States of America; University of Wisconsin-Madison, UNITED STATES

## Abstract

White-nose syndrome (WNS) in North American bats is caused by an invasive cutaneous infection by the psychrophilic fungus *Pseudogymnoascus destructans* (*Pd*). We compared transcriptome-wide changes in gene expression using RNA-Seq on wing skin tissue from hibernating little brown myotis (*Myotis lucifugus*) with WNS to bats without *Pd* exposure. We found that WNS caused significant changes in gene expression in hibernating bats including pathways involved in inflammation, wound healing, and metabolism. Local acute inflammatory responses were initiated by fungal invasion. Gene expression was increased for inflammatory cytokines, including interleukins (IL) IL-1β, IL-6, IL-17C, IL-20, IL-23A, IL-24, and G-CSF and chemokines, such as Ccl2 and Ccl20. This pattern of gene expression changes demonstrates that WNS is accompanied by an innate anti-fungal host response similar to that caused by cutaneous *Candida albicans* infections. However, despite the apparent production of appropriate chemokines, immune cells such as neutrophils and T cells do not appear to be recruited. We observed upregulation of acute inflammatory genes, including prostaglandin G/H synthase 2 (cyclooxygenase-2), that generate eicosanoids and other nociception mediators. We also observed differences in *Pd* gene expression that suggest host-pathogen interactions that might determine WNS progression. We identified several classes of potential virulence factors that are expressed in *Pd* during WNS, including secreted proteases that may mediate tissue invasion. These results demonstrate that hibernation does not prevent a local inflammatory response to *Pd* infection but that recruitment of leukocytes to the site of infection does not occur. The putative virulence factors may provide novel targets for treatment or prevention of WNS. These observations support a dual role for inflammation during WNS; inflammatory responses provide protection but excessive inflammation may contribute to mortality, either by affecting torpor behavior or causing damage upon emergence in the spring.

## Author Summary

White-nose syndrome is the most devastating epizootic wildlife disease of mammals in history, having killed millions of hibernating bats in North America since 2007. We have used next-generation RNA sequencing to provide a survey of the gene expression changes that accompany this disease in the skin of bats infected with the causative fungus. We identified possible new mechanisms that may either provide protection or contribute to mortality, including inflammatory immune responses. Contrary to expectations that hibernation represents a period of dormancy, we found that gene expression pathways were responsive to the environment. We also examined which genes were expressed in the pathogen and identified several classes of genes that could contribute to the virulence of this disease. Gene expression changes in the host were associated with local inflammation despite the fact that the bats were hibernating. However, we found that hibernating bats with white-nose syndrome lack some of the responses known to defend other mammals from fungal infection. We propose that bats could be protected from white-nose syndrome if these responses could be established prior to hibernation or if treatments could block the virulence factors expressed by the pathogen.

## Introduction

White-nose syndrome (WNS) is an epizootic disease that has killed millions of bats in North America [1, 2]. WNS is caused by the psychrophile *Pseudogymnoascus destructans* (*Pd*) (formerly *Geomyces destructans*), an ascomycete fungal pathogen [3–5] that affects bats during hibernation. *Pd* grows at temperatures between 2 and 18°C and can infect bats while they hibernate [4, 6]. *Pd* is invasive and damages the cutaneous tissues of bats, including the wing [7], forming characteristic cupping erosions that are diagnostic of *Pd* infection [8]. Mortality rates due to WNS vary by species. In the little brown myotis, *Myotis lucifugus*, the mortality rate is up to 91% in affected caves [9, 10] whereas WNS resistance has been reported in the big brown bat, *Eptesicus fuscus* [11]. Bats in Europe are exposed to endemic *Pd*, but do not exhibit WNS mortality and appear to be resistant to the disease [12], despite cutaneous invasion by *Pd* [13].

Cutaneous infection by *Pd* causes some species of bats to arouse more frequently from torpor [5, 14, 15]. Although hibernating mammals spend less than 1% of their time euthermic [16], they use up to 90% of their stored energy during these periods [17, 18]. Because each arousal in little brown myotis utilizes an estimated 108 mg of stored fat [18], the increase in arousal frequency caused by WNS explains 58% of the morbidity rate associated with *Pd* infection [14]. Other factors that are also associated WNS pathology include effects of *Pd* infection on the integrity of wing tissue [7, 19], electrolyte balance and hydration [7, 20, 21], chronic respiratory acidosis [22], oxidative stress [23], and immune function [24]. The relative importance of each of these mechanisms in causing death in WNS is not clear, and the most likely model that has emerged is a multi-stage progression of WNS with contributions of several of these factors [22]. Differences in susceptibility to WNS between species in North America may be explained in part by different responses to *Pd* infection such as changes in thermoregulatory behavior. Understanding host responses to *Pd* infection may provide insight that could be useful for improving survival of affected species.

Cutaneous fungal infections in mammals are first recognized by components of the innate immune system, including C-type lectin receptors and Toll-like receptors [25]. Conserved components of the fungal cell wall activate pattern recognition receptors on phagocytes such as neutrophils, macrophages, and dendritic cells, and on epithelial cells [26]. Activation of these cells can lead to induction of the inflammasome, the production of inflammatory cytokines, and generation of reactive oxygen species that can mediate fungal cell killing [25]. The importance of the innate immune response to the initial recognition of fungal infections is demonstrated by the observation that deficiencies in these signaling pathways can lead to chronic fungal infections in humans [27, 28]. In the absence of invasion, colonization by commensal fungi can be maintained through tolerance mechanisms mediated by interactions with dendritic cells and epithelial cells in the skin [29]. Local activation of innate immune pathways can slow the growth of invasive pathogenic fungi and promote tolerance, possibly leading to a commensal relationship with the fungus [30], but is not usually sufficient to clear infections. Clearance of infections typically requires T helper (Th) cells, as demonstrated by the susceptibility of patients with acquired immune deficiency syndrome, immunosuppressant therapy, or chemotherapy to fungal infections [31]. These T cell responses can be mediated by Th17 cells [32, 33] or, in some cases, Th1 cells [34], with Th2 responses typically associated with greater susceptibility [35]. Th17 responses can contribute to clearance of invasive fungal infections through the actions of IL-17A and IL-22 [36] and the further recruitment and activation of neutrophils [37]. These T cell subsets have not been well characterized in bats, but those T-cell mediated immune mechanisms that have been studied appear to be conserved between bats and other mammals [38–41].

Fungal infections in animals are typically life-threatening only upon suppression of adaptive immune responses in the host, such as when chytrid fungus (*Batrachochytrium dendrobatidis*) blocks lymphocyte-mediated inflammatory responses [42]. Hibernation produces a natural suppression of some immune responses in mammal species where it has been studied. During hibernation, when the conservation of energy is critical, certain immunological mechanisms are downregulated while others remain unaffected [43–51]. Changes during hibernation can include depressed antibody responses [44, 52], decreased ability of T and B lymphocytes to proliferate in response to challenge [53, 54], and reduced complement activity [47]. Hibernation does not affect all immune responses equally, as shown in thirteen-lined ground squirrels (*Ictidomys tridecemlineatus*) that have a suppressed T-independent antibody response but are capable of mounting a T cell-dependent response during hibernation [44]. Studies of transcriptome-wide changes during hibernation in squirrels [55–59] have shown expression changes in genes involved in metabolism, oxidative stress, protein folding, ischemia/hypoxia, and other processes, but these studies were not examining an active immune response. Hibernation is also known to affect the distribution of leukocytes [45, 60] and platelets [61]. However, we have an incomplete understanding of how hibernation affects the suppression, or subsequent recovery, of immune responses [43], or how immune physiology in bats during hibernation may differ from that of rodents.

The cost of immune suppression during torpor is presumably outweighed by the benefits of energy conservation because most pathogens are not capable of proliferating at the low body temperatures of hibernating animals. However, the psychrophilic nature of *Pd* allows it to infect bats within hibernacula [2, 4]. The brief euthermic bouts of hibernating bats are shorter than most other hibernating mammalian species [14, 62] and it may not be possible for a bat naïve to a pathogen to mount a primary immune response in the few hours that it is euthermic throughout the hibernation season. We have observed antibody responses to *Pd* in bats, but these responses are strongest in active bats exposed to *Pd* after emergence from hibernation [63]. Therefore, hibernating bats may keep pathogens in check by relying on hypothermia, innate immune responses, and/or memory immune responses. The psychrophilic nature of *Pd* overcomes the first of these barriers to infection and the difficulty in fighting fungal pathogens with innate mechanisms alone may allow *Pd* to proliferate and invade the cutaneous tissues of bats.

The WNS panzootic has created an urgent need to understand if North American bat populations can persist in the presence of the fungal pathogen [1, 10]. Understanding the complete array of host responses mounted by bats afflicted with WNS may help illuminate sources of variation in survival within and among bat species. To determine which host responses are activated by *Pd* infection, we measured transcriptome-wide gene expression levels in bat wing tissue from hibernating bats affected by WNS. Gene expression was compared to bats that were hibernated in captivity in the absence of *Pd* exposure. We hypothesized that *Pd* infection would cause changes in gene expression that would reveal physiological responses during WNS that might be either protective or pathological. By using next-generation RNA sequencing to examine transcriptome-wide gene expression changes we expected to discover consistent patterns of host responses that occur in *Pd*-infected tissues. Combined with changes in gene expression within the *Pd* pathogen, these results have provided a survey of the host and pathogen interactions occurring during WNS.

## Results

### Gene Expression Changes Revealed by Next Generation RNA Sequencing

To determine the host response mounted by little brown myotis to *Pd* during hibernation, we measured changes in gene expression at the whole transcriptome level. Wing tissue samples were obtained from hibernating little brown myotis with no known exposure to *Pd* and bats exhibiting physical signs of WNS, as shown in Table 1. Histopathology [8] and quantitative PCR (qPCR) for *Pd* [64] were used to confirm the WNS status of each bat (Table 1). Cupping erosions diagnostic of WNS were found on all 6 bats captured in Kentucky, but on none of the 5 bats from states negative for WNS at the time of capture. Low levels of neutrophilic inflammation were found in all 11 wing samples (Table 1; Infl), although this inflammation was not associated with sites of *Pd* infection. All 6 WNS-affected bats tested positive for *Pd* by qPCR, although the fungal load measured on wing swabs (Table 1; qPCR) did not correlate with the number of cupping erosions found by histology (Table 1; WNS). As previously shown [5, 14, 15], WNS-affected bats had significantly lower body condition (Table 1; SMI; p = 0.017, t = 2.9255, df = 9).

**Table 1:** Samples used for next generation RNA sequencing.

Sample	Location	Date Captured	Date Sampled	Sex	Mass	SMI^1^	*Pd* Load by qPCR^2^	Histology	
								WNS^3^	Infl^4^
MI011	Mine in Dickinson Co, MI	5-Nov-2011	22-Mar-2012	M	6.70	6.69	Negative	0	9
MN064	Mine in Saint Louis Co, MN	16-Nov-2011	22-Mar-2012	F	7.41	7.15	Negative	0	2
MN075	Mine in Saint Louis Co, MN	16-Nov-2011	22-Mar-2012	F	7.47	7.68	Negative	0	50
MN090	Mine in Saint Louis Co, MN	16-Nov-2011	22-Mar-2012	M	7.66	7.51	Neg/Pos?	0	13
IL114	Mine in LaSalle Co, IL	17-Nov-2011	22-Mar-2012	F	7.13	7.40	Negative	0	2
KY06	Cave 1 in Breckinridge Co, KY	12-Mar-2014	12-Mar-2014	F	6.04	6.17	120 000	352	25
KY07	Cave 1 in Breckinridge Co, KY	12-Mar-2014	12-Mar-2014	F	6.90	6.89	13 000	438	57
KY11	Cave 1 in Breckinridge Co, KY	12-Mar-2014	12-Mar-2014	M	5.47	6.12	169 000	288	3
KY19	Cave 2 in Breckinridge Co, KY	12-Mar-2014	12-Mar-2014	M	6.28	6.93	64 000	117	9
KY23	Cave 2 in Breckinridge Co, KY	12-Mar-2014	12-Mar-2014	F	6.58	6.89	21 000	234	10
KY39	Cave in Jackson Co, KY	13-Mar-2014	13-Mar-2014	M	6.28	6.69	120 000	197	10

^1^ Scaled mass index: (mass(in g))*(38.01/(forearm length(in mm))^1.406

^2^ Wing swabs from MI, MN, and IL were measured in duplicate and determined to be positive for *Pd* if the cycle-threshold was less than 40. Samples from KY were quantified in *Pd* genomic equivalents relative to swabs spiked with 10 000 *Pd* conidia.

^3^ Cupping erosions characteristic of WNS per roll of wing tissue.

^4^ Foci of neutrophilic inflammation per roll of wing tissue.

Next generation RNA sequencing (RNA-Seq) was performed using poly-A selected RNA isolated from each RNAlater-preserved wing tissue sample (S1 Table). Using expression levels of *Pd*-derived transcripts, we confirmed that all 6 WNS-affected bats had abundant expression of *Pd* genes. The *Pd*-derived transcripts were not present at significant levels in any of the 5 samples from unaffected bats (S2 Table; p = 2.2x10^-6^, t = 21.5, df = 5.33), including the MN090 sample that had tested positive for *Pd* by qPCR in one of the two replicates (Table 1). Because high levels of differential expression of *Pd* transcripts would make it more difficult to detect significant changes in host gene expression, the assembly was filtered [65] to remove *Pd*-derived sequences. Comparison of the filtered assembly with the original revealed that removing the *Pd* sequences did not significantly decrease the completeness of the assembly (S3 Table) as determined by BUSCO [66]. This filtered assembly (S1 Dataset) was used to calculate differential expression in host genes between the unaffected and WNS-affected samples.

**Fig 1:**
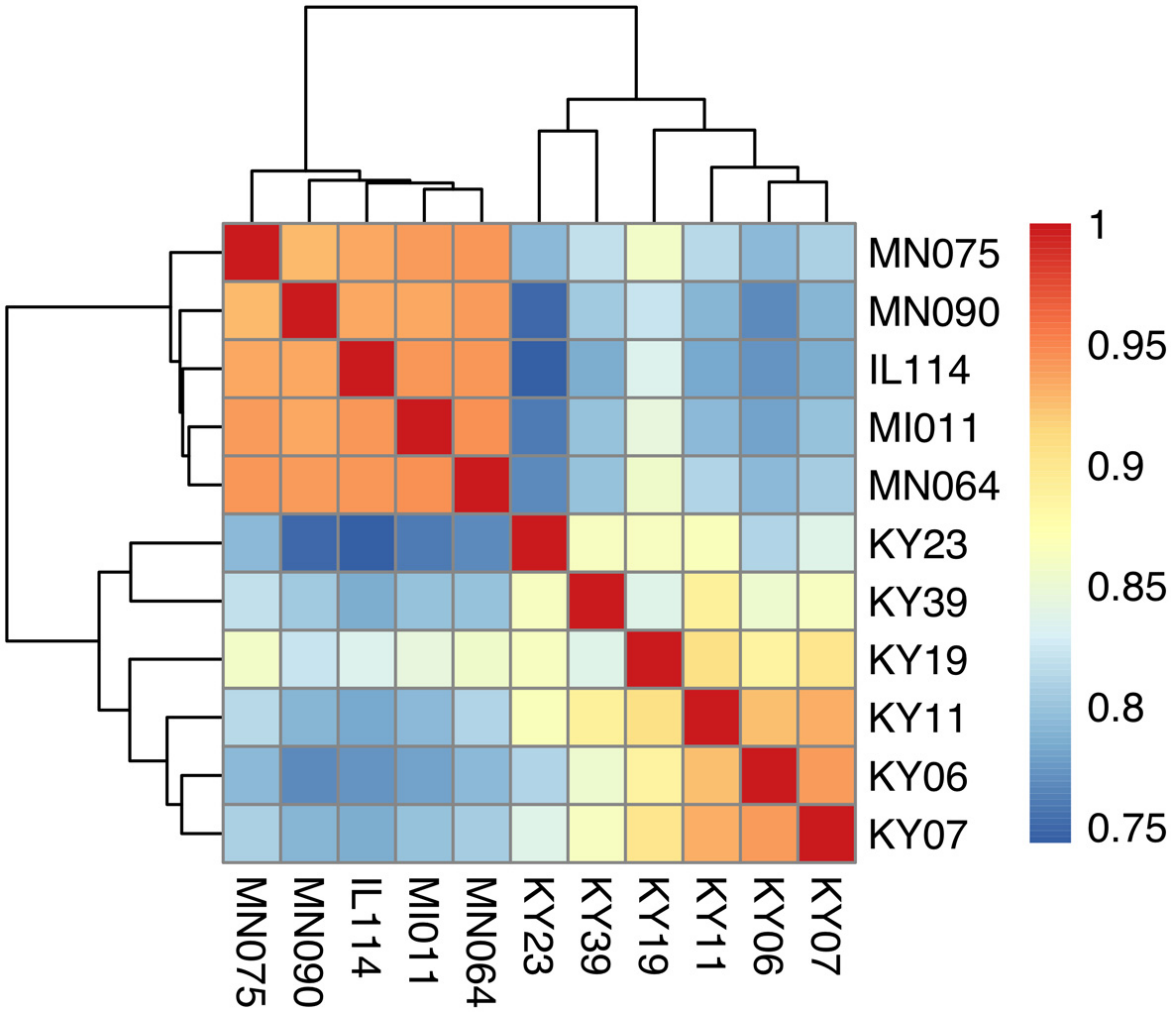
Hierarchical clustering of gene expression in WNS-affected and unaffected bats. Hierarchical clustering of differentially expressed genes using Pearson correlation complete-linkage clustering. Scale shows Pearson correlation coefficient.

We compared host gene expression across all samples (S2 Dataset) using DESeq2 [67] to identify transcript clusters that were expressed at a minimum of 2-fold difference and significant at a false discovery rate (FDR) of 0.05 (S1 Fig). We found 1804 transcript clusters that were expressed at higher levels, and 1925 transcript clusters expressed at lower levels, in WNS-affected bat tissues (S4 Table). Hierarchical clustering (Fig 1) revealed that expression of these transcripts from all 5 bats without WNS was similar to each other. Gene expression in wing tissue from WNS-affected bats was different from unaffected bats and more similar to each other, as predicted. The normalized expression levels of the 3729 identified transcript clusters differentially expressed are listed in S4 Table.

Differential expression of individual gene isoforms was further analyzed using EBSeq [68], an empirical Bayesian approach to modeling gene expression. For each transcript cluster identified as differentially expressed by DESeq2, we used EBSeq to determine if any of the individual transcripts were differentially expressed at a posterior probability greater than 0.99 (S4 Table). Of the 3729 differentially expressed transcript clusters identified by DESeq2, EBSeq identified at least one differentially expressed transcript for 1427 (38% of total, 43% of upregulated genes and 33% of downregulated genes). These results indicate that differences in gene expression are likely due to alternative splicing or other isoform differences for many of the differentially expressed genes.

To annotate the functions of these genes and identify those likely to be involved with host responses to *Pd* infection, we used the Trinotate pipeline. BLAST was used to identify 1365 upregulated transcripts and 325 downregulated transcripts in WNS-affected tissues with significant homology to known genes from vertebrates in the Swissprot database. Of the 2295 remaining transcripts, 13 were mapped to genes from non-vertebrates in the Swissprot database, presumably due to environmental contamination or incomplete removal of *Pd* transcript sequences. Of the 2842 trinity transcript clusters without a BLASTx match in Swissprot, 2731 (96%) were found to align to sequences (e-value < 0.0001) in the little brown myotis genome. Of the aligned transcripts, 204 (7.4%) were found to correspond to previously identified non-coding RNA sequences. Of the 111 transcript clusters without a transcript that aligned to the little brown myotis genome or Swissprot, BLAST was used to align their transcripts to the UniRef90 database. We found that 7 genes aligned to vertebrate homologs, 9 aligned to fungal homologs, and 15 aligned to other metagenomic sequences. We were unable to identify homologous sequences for any transcripts from 80 (2.1%) of the transcript clusters that were differentially expressed.

**Fig 2:**
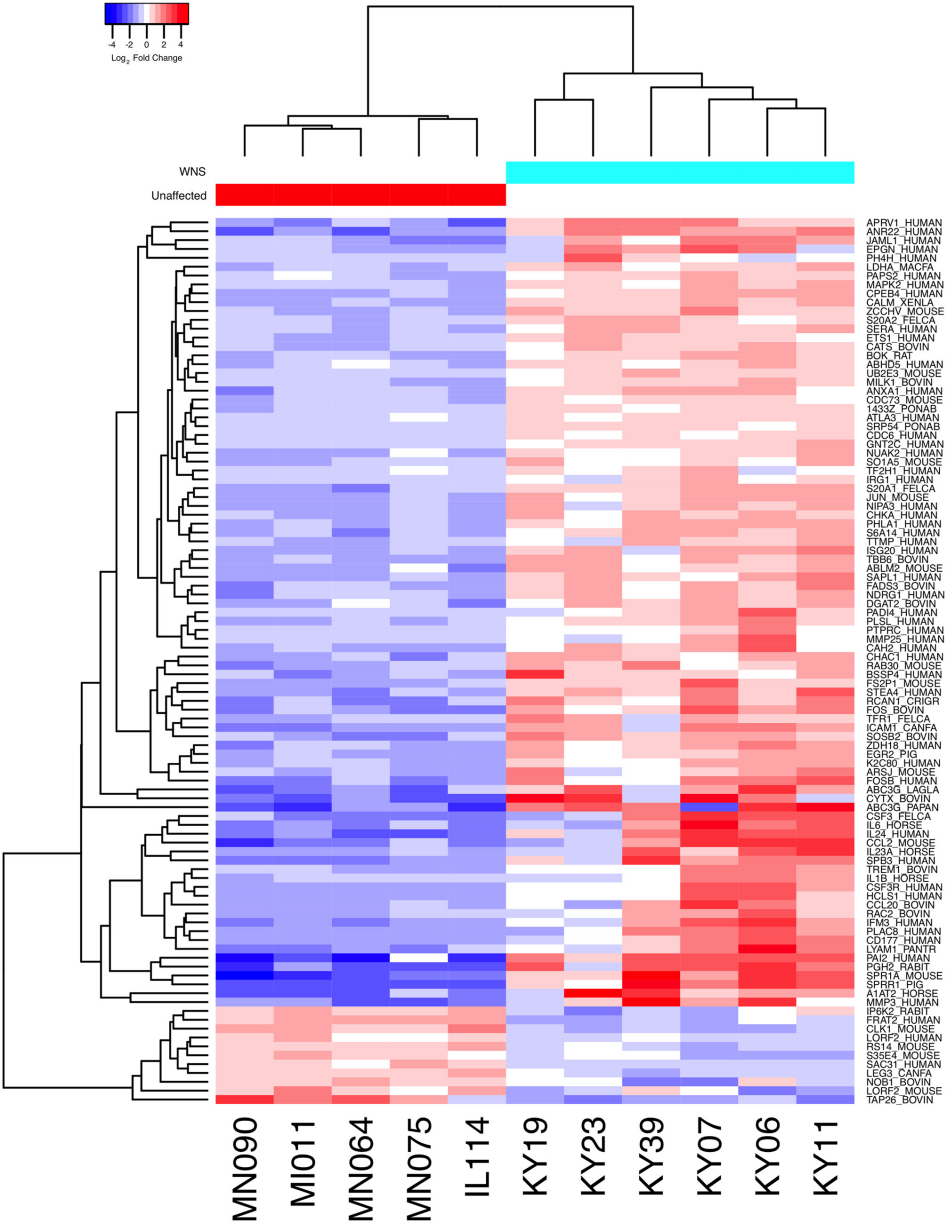
Global transcriptional analysis of WNS-affected and unaffected bats by RNA-Seq. Centered log_2_ fold changes are shown for the 100 most significant differentially expressed identified genes. Adjusted p values ranged from 3.3x10^-5^ to 2.8x10^-18^. The heatmap of TMM-normalized FPKM expression estimates is centered and log_2_ scaled from a minimum of -4.8 to a maximum of 4.8. Transcripts were identified by BLAST alignment to the SwissProt database.

Expression levels for the Swissprot-identified transcript clusters with the 100 lowest adjusted p values are shown in Fig 2 (see S4 Table for all results). Some of the differentially expressed genes with putative functions that were predicted to associate with host responses to a fungal pathogen are listed in Table 2. WNS caused dramatic changes in expression of genes involved in inflammation, immune responses, wound healing, metabolism, and oxidative stress, even though the bats were hibernating during the *Pd* infection. Most of these genes were upregulated in WNS-affected tissues, while a much smaller number of identified genes with putative functions in these categories were downregulated (Tables 2 and S4).

**Table 2:** Selected genes differentially expressed in WNS-affected tissues.

Gene^1^	Full Name	FC^2^	pvalue^3^	padj^4^	EB FC^5^	PPEE^6^
**Inflammation**						
IL23A	Interleukin-23 subunit alpha	32.6	4.8E-09	2.0E-06	33.6	1.9E-08
PGH2	Prostaglandin G/H synthase 2 (Cyclooxygenase-2)	30.5	1.2E-14	2.1E-11	42.3	3.2E-12
IL6	Interleukin-6	30.3	1.1E-10	9.9E-08	50.9	5.2E-09
MMP25	Matrix metalloproteinase-25	25.7	5.1E-09	2.0E-06	25.5	5.7E-06
CSF3R	Granulocyte colony-stimulating factor receptor	23.7	1.7E-09	9.1E-07	31.3	6.9E-08
CCL20	C-C motif chemokine 20	22.0	1.4E-07	3.0E-05	21.1	3.3E-06
IL20	Interleukin-20	20.2	2.9E-07	5.3E-05	21.5	1.5E-06
CSF3	Granulocyte colony-stimulating factor	19.1	6.8E-08	1.8E-05	44.4	2.3E-07
IL1B	Interleukin-1 beta	17.7	5.6E-08	1.5E-05	13.6	6.6E-06
IL1A	Interleukin-1 alpha	15.4	4.5E-05	2.1E-03	11.4	3.1E-04
PA21	Phospholipase A2	13.8	3.7E-04	9.1E-03	NA	NA
CCL2	C-C motif chemokine 2	12.3	6.5E-06	5.3E-04	16.7	2.7E-05
IL17C	Interleukin-17C	10.3	6.5E-07	9.6E-05	12.7	3.0E-05
IL19	Interleukin-19	9.0	1.4E-04	4.7E-03	NA	NA
IL24	Interleukin-24	7.6	3.4E-08	1.0E-05	18.9	1.3E-05
NCF2	Neutrophil cytosol factor 2	5.7	9.9E-05	3.8E-03	5.0	5.6E-04
PG12A	Group XIIA secretory phospholipase A2	2.7	9.4E-07	1.3E-04	3.1	5.7E-07
S10AC	Protein S100-A12	2.3	9.0E-04	1.7E-02	NA	NA
**Other Immune Genes**						
ABC3G	DNA dC->dU-editing enzyme APOBEC-3G	32.4	3.9E-12	4.7E-09	54.2	4.1E-10
LIRA6	Leukocyte immunoglobulin-like receptor subfamily A member 6	23.1	1.5E-05	9.5E-04	NA	NA
HPT	Haptoglobin	18.9	3.3E-05	1.7E-03	72.2	3.9E-08
CD3G	T-cell surface glycoprotein CD3 gamma chain	14.8	6.7E-06	5.5E-04	NA	NA
CLC4D	C-type lectin domain family 4 member D	12.5	7.0E-05	2.9E-03	NA	NA
PTPRC	Receptor-type tyrosine-protein phosphatase C	12.3	1.4E-07	3.1E-05	16.0	1.1E-04
CLC4E	C-type lectin domain family 4 member E	12.3	3.6E-07	6.0E-05	NA	NA
CLC7A	C-type lectin domain family 7 member A	10.9	5.4E-07	8.3E-05	NA	NA
CO3	Complement C3	10.1	2.7E-03	3.4E-02	64.9	8.0E-08
TLR9	Toll-like receptor 9	8.9	1.4E-06	1.7E-04	6.2	2.5E-05
S10A3	Protein S100-A3	8.4	4.8E-04	1.1E-02	NA	NA
CLC6A	C-type lectin domain family 6 member A	7.0	1.5E-04	5.0E-03	7.0	3.9E-03
CLC1A	C-type lectin domain family 1 member A	6.5	2.4E-06	2.6E-04	NA	NA
D103A	Beta-defensin 103A	6.1	5.4E-06	4.7E-04	6.3	3.2E-03
CLC5A	C-type lectin domain family 5 member A	5.1	3.3E-04	8.3E-03	NA	NA
BIRC3	Baculoviral IAP repeat-containing protein 3	3.4	7.9E-05	3.2E-03	4.6	3.3E-04
UNG	Uracil-DNA glycosylase	-3.8	1.0E-03	1.8E-02	NA	NA
LEG3	Galectin-3	-3.4	5.0E-08	1.4E-05	0.28	1.0E-10
**Wound Healing**						
SPRR1	Cornifin	184.6	4.1E-17	1.0E-13	66.4	< 1E-16
LCE3C	Late cornified envelope protein 3C	17.5	2.1E-09	1.1E-06	15.0	6.2E-05
FIBB	Fibrinogen beta chain	15.9	1.8E-04	5.6E-03	67.2	5.2E-08
FIBA	Fibrinogen alpha chain	12.4	1.1E-03	1.9E-02	88.1	1.6E-08
ARGI1	Arginase-1	11.9	4.8E-04	1.1E-02	NA	NA
FIBG	Fibrinogen gamma chain	11.8	1.4E-03	2.2E-02	48.9	1.6E-07
EPGN	Epigen	10.3	2.0E-09	1.1E-06	13.1	7.3E-06
EREG	Proepiregulin	8.8	6.9E-07	1.0E-04	10.7	4.8E-04
KLK6	Kallikrein-6	8.1	3.4E-07	5.9E-05	9.1	4.2E-04
K1C17	Keratin, type I cytoskeletal 17	5.9	1.3E-05	8.7E-04	6.6	3.7E-03
P63	Tumor protein 63	3.1	2.0E-05	1.2E-03	16.9	9.8E-05
**Metabolism**						
PLAC8	Placenta-specific gene 8 protein	37.0	1.5E-09	8.4E-07	25.9	1.3E-05
LIPP	Pancreatic triacylglycerol lipase	22.3	1.0E-06	1.3E-04	19.4	2.7E-06
ANGL3	Angiopoietin-related protein 3	17.2	1.5E-04	4.9E-03	NA	NA
APOC4	Apolipoprotein C-IV	16.1	7.9E-05	3.2E-03	NA	NA
APOC3	Apolipoprotein C-III	15.8	1.8E-04	5.5E-03	42.5	8.7E-07
APOC2	Apolipoprotein C-II	14.5	3.7E-04	9.2E-03	45.0	6.8E-07
FFAR2	Free fatty acid receptor 2	7.7	1.8E-05	1.1E-03	7.3	9.1E-04
HCAR2	Hydroxycarboxylic acid receptor 2	4.0	6.9E-04	1.4E-02	NA	NA
IP6K2	Inositol hexakisphosphate kinase 2	-5.0	3.8E-06	3.6E-04	0.19	2.2E-03
ACACA	Acetyl-CoA carboxylase 1	-4.2	1.1E-03	1.9E-02	0.16	6.7E-03
**Other Oxidative Stress**						
MMP3	Stromelysin-1	34.0	3.4E-10	2.5E-07	57.7	6.6E-08
PERT	Thyroid peroxidase	3.7	2.7E-03	3.4E-02	NA	NA
PRDX2	Peroxiredoxin-2	3.6	1.1E-05	7.8E-04	NA	NA
HMOX1	Heme oxygenase 1	3.0	5.4E-04	1.2E-02	8.2	1.7E-03

^1^ BLAST hit with the lowest E-value in the Swissprot database. Only genes with E < 1E-05 were considered.

^2^ Fold change in gene expression of the WNS-affected samples compared to the unaffected samples as determined by DESeq2. Negative values indicate higher expression in the unaffected samples.

^3^ Probability of differential expression determined by DESeq2.

^4^ Adjusted probability of differential expression after Benjamini-Hochberg FDR correction.

^5^ Posterior probability fold change in EBSeq-estimated expression of each transcript in WNS-affected tissues over unaffected tissues. NA indicates that no isoform for that gene was differentially expressed at an FDR < 0.001

^6^ Posterior probability estimate by EBSeq that the isoform is differentially expressed.

**Fig 3:**
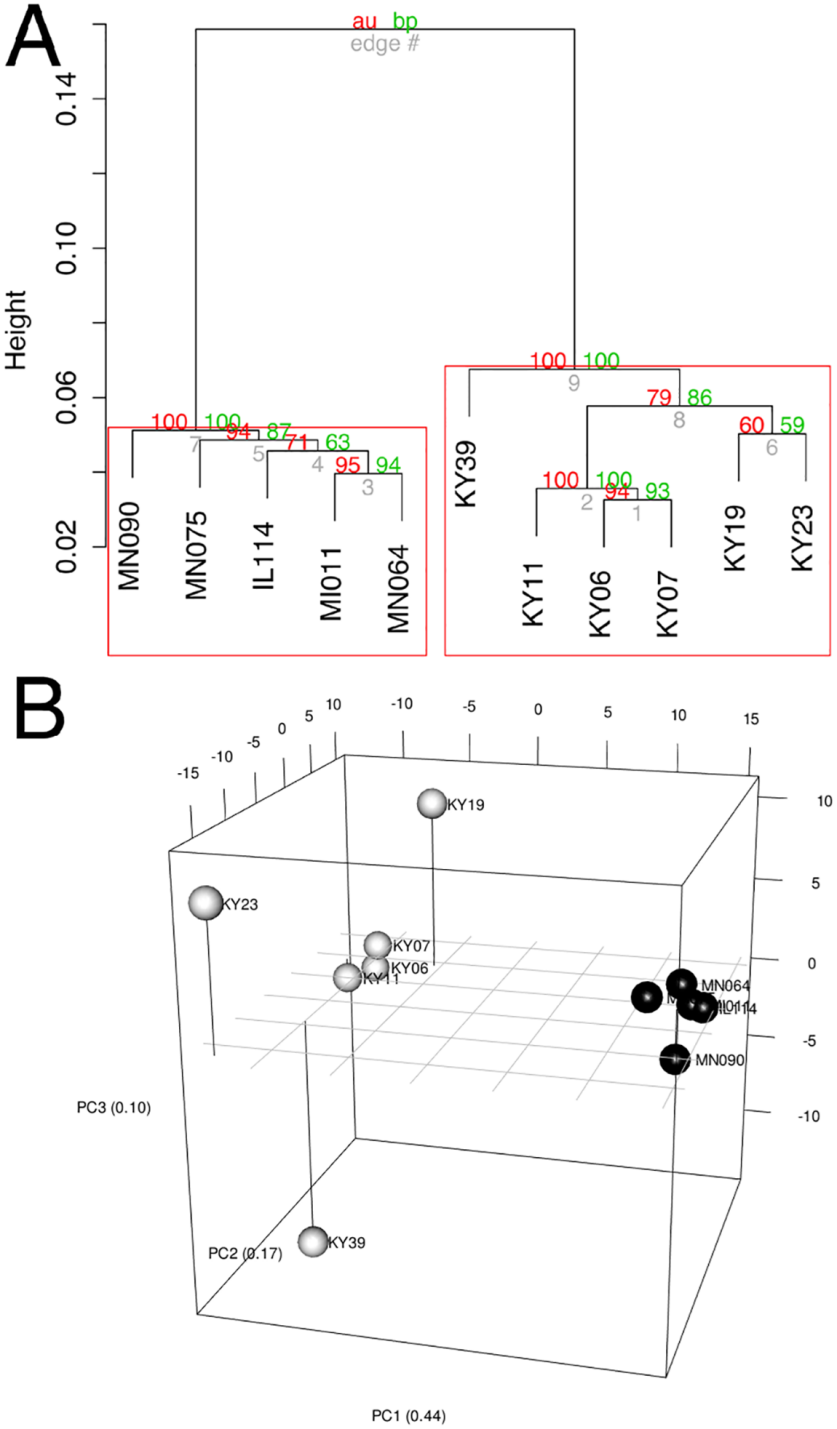
Clustering of gene expression patterns. (A) Bootstrap analysis of regularized-log transformed counts to assign confidence levels to sample clustering. Red values are approximately unbiased p values and green values are bootstrap probabilities. Red boxes indicate clusters supported at a confidence level of 99%. (B) Principal component analysis of regularized-log transformed counts of differentially expressed genes. Black spheres represent unaffected bats and white spheres represent WNS-affected bats.

To determine if all 6 little brown myotis with WNS exhibit similar changes in gene expression, we performed clustering analysis of the differentially expressed transcripts (Fig 1). To confirm the significance of these patterns of gene expression, bootstrap analysis of clustering was performed [69]. The clustering of the unaffected samples together and the clustering of the WNS-affected samples together was verified with a confidence of 99% (Fig 3A). Principal component analysis was performed to better understand the relationships between the transcripts expressed in the 11 samples (Fig 3B). All 5 samples from unaffected bats were very similar based on the first three principal components identified, which account for 71% of the variance in these transcripts. The WNS-affected bat samples have more diverse gene expression (S5 Table) and PC1 (accounting for 44% of the variance) differentiates all 6 from the unaffected bat samples. The genes represented by PC1 include those that are more highly expressed in unaffected than WNS-affected wing tissue (Fig 2). PC2 (17% of the variance) and PC3 (10% of the variance) distinguish the KY19, KY23, and KY39 samples from the other two WNS-affected samples and from the unaffected samples. The rotation values of principal component analysis (S5 Table) reveal that inflammatory genes made the greatest contribution to PC2. Clustering analysis revealed diverse host responses among the bats infected with *Pd*.

### Metabolic and Inflammatory Immune Pathways Associated with WNS

**Fig 4:**
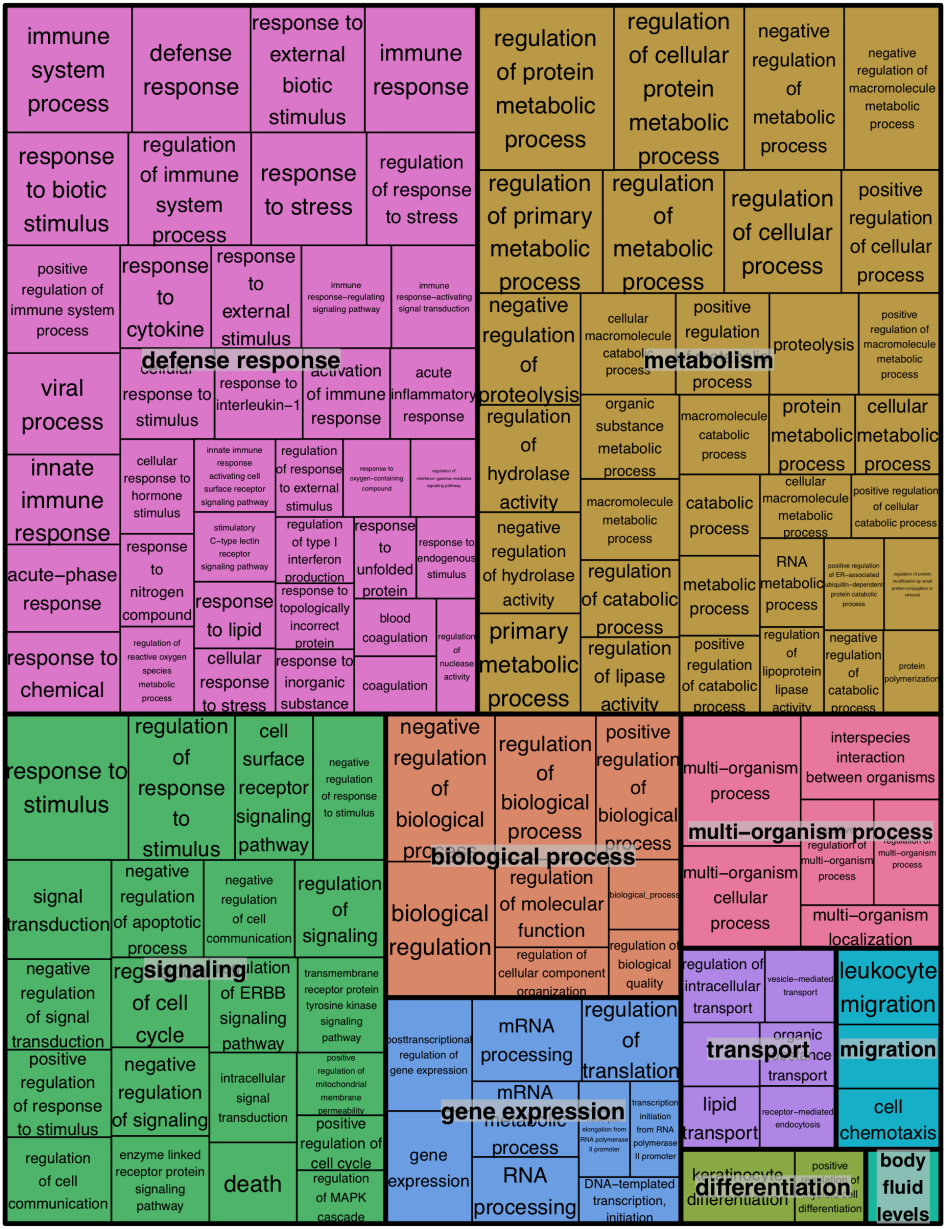
REVIGO treemap summarizing gene ontology biological process categories over-represented in WNS-affected tissues. GOrilla was used to identify Gene Ontology Biological Processes that were over-represented among transcripts more highly expressed in WNS-affected tissues at an FDR cutoff of 0.1 (S6 Table). Over-represented categories with p values of less than 0.001 (290 terms) were used to generate a treemap colored by functional category. The size of each rectangle is proportional to the p value for that category.

We next examined the functional pathways that were most affected in little brown myotis infected with *Pd*. For this gene ontology analysis, DESeq2 results on transcript isoforms were used with a higher FDR threshold of 0.1, as is typical for this type of analysis. From WNS-affected bat tissue, 3104 upregulated transcripts were aligned with BLAST to the human Uniprot database. Homologs for these transcripts were identified and a list of 1937 unique Ensembl IDs associated with upregulated genes was generated (S6 Table). GOrilla [70] was used to determine significantly upregulated gene ontology categories from the Uniprot GO ID database (Table 3 and S7 Table) and REVIGO [71] was used to visualize biological processes that were significantly overrepresented in the WNS-affected transcriptome (Fig 4). The functional analysis revealed that *Pd* infection increases expression of genes involved in metabolism, defense responses, and other pathways (Table 3).

**Table 3:** Selected over-represented gene ontology biological process categories.

Category	GO: Biological Process Term	p^1^	FDR^2^	Enrich^3^	# DE^4^	# Cat^5^
0051246	regulation of protein metabolic process	4.2E-15	5.3E-11	1.46	344	1810
0006952	defense response	2.3E-10	2.2E-07	1.56	179	881
0051248	negative regulation of protein metabolic process	4.3E-10	2.9E-07	1.61	156	746
0080134	regulation of response to stress	9.9E-09	4.6E-06	1.46	194	1019
0044403	symbiosis, encompassing mutualism through parasitism	3.4E-08	1.2E-05	1.64	113	528
0006953	acute-phase response	3.5E-07	8.9E-05	4.11	15	28
0034097	response to cytokine	1.0E-06	2.1E-04	1.88	58	237
0045089	positive regulation of innate immune response	1.1E-06	2.1E-04	1.89	57	232
0006954	inflammatory response	1.1E-06	2.1E-04	1.81	64	271
0030216	keratinocyte differentiation	3.8E-06	5.4E-04	3.14	18	44
0070555	response to interleukin-1	7.5E-06	9.5E-04	2.92	19	50
0002526	acute inflammatory response	8.4E-06	1.0E-03	3.11	17	42
0002755	MyD88-dependent toll-like receptor signaling pathway	1.7E-05	1.8E-03	2.56	22	66
0034162	toll-like receptor 9 signaling pathway	4.1E-05	3.5E-03	2.56	20	60
0050860	negative regulation of T cell receptor signaling pathway	5.7E-05	4.5E-03	4.72	8	13
2000378	negative regulation of reactive oxygen species metabolic process	8.7E-05	6.3E-03	3.52	11	24
0009913	epidermal cell differentiation	9.0E-05	6.5E-03	2.44	20	63
0030593	neutrophil chemotaxis	1.3E-04	9.1E-03	3.02	13	33
0002223	stimulatory C-type lectin receptor signaling pathway	2.3E-04	1.4E-02	2.01	27	103
0050878	regulation of body fluid levels	2.7E-04	1.6E-02	1.43	88	472
0051005	negative regulation of lipoprotein lipase activity	2.9E-04	1.7E-02	7.68	4	4
0032480	negative regulation of type I interferon production	3.7E-04	2.1E-02	2.77	13	36

^1^ Over-represented p value.

^2^ False discovery rate after Benjamini-Hochberg adjustment for multiple comparisons.

^3^ Enrichment of differentially expressed genes in this category.

^4^ Number of differentially expressed genes in this category identified by GOrilla at a p value cutoff of 0.001.

^5^ Number of genes in this GO category represented in the background set.

For the transcripts that showed lower expression in WNS-affected tissue (Fig 2), the same analysis was performed. Of the 1152 identified transcripts that were downregulated at an FDR of 0.1, 694 homologous human genes were identified by BLAST and mapped to Ensembl gene IDs (S6 Table). GOrilla did not identify any Biological Process categories that were significantly downregulated in the WNS-affected bat tissue.

### Host-Pathogen Interactions during WNS

**Fig 5:**
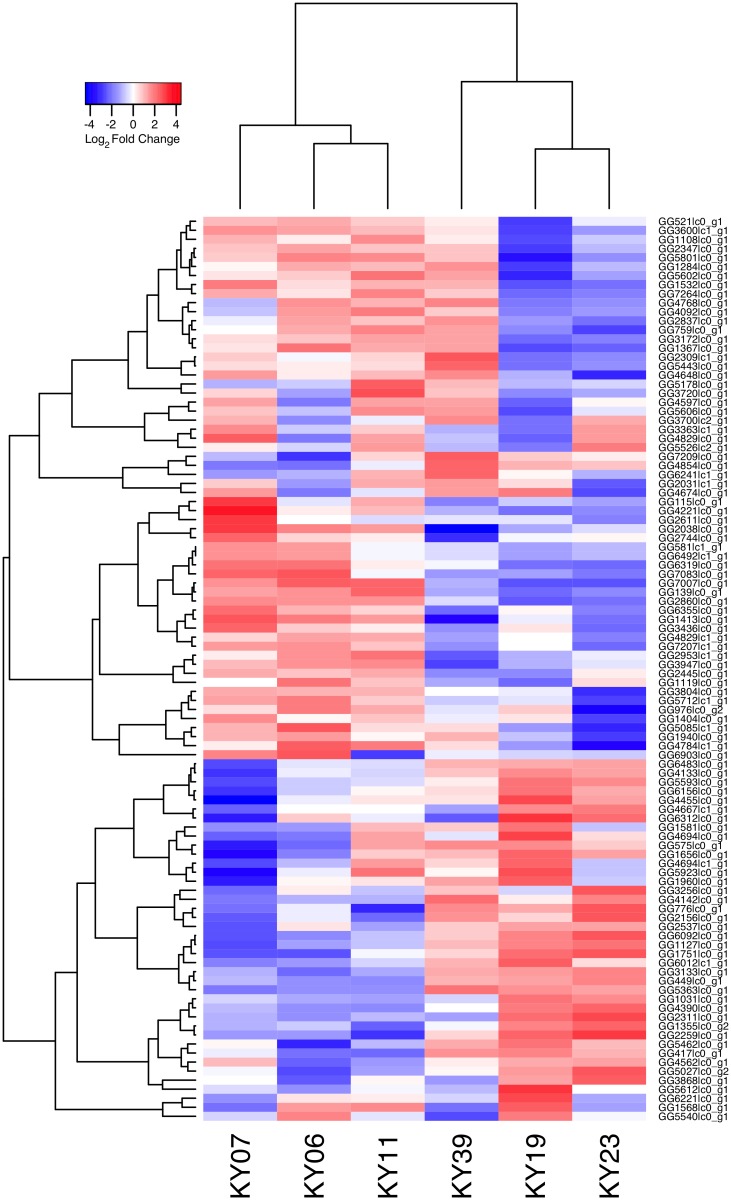
Transcriptional analysis of *Pd* gene expression on bats with WNS. Centered log_2_ fold changes are shown for 100 *Pd* genes with the greatest variance and a minimum TMM-normalized FPKM expression of 1 in all 6 samples. The heatmap is scaled from a minimum of -4.4 to a maximum of 4.4.

**Fig 6:**
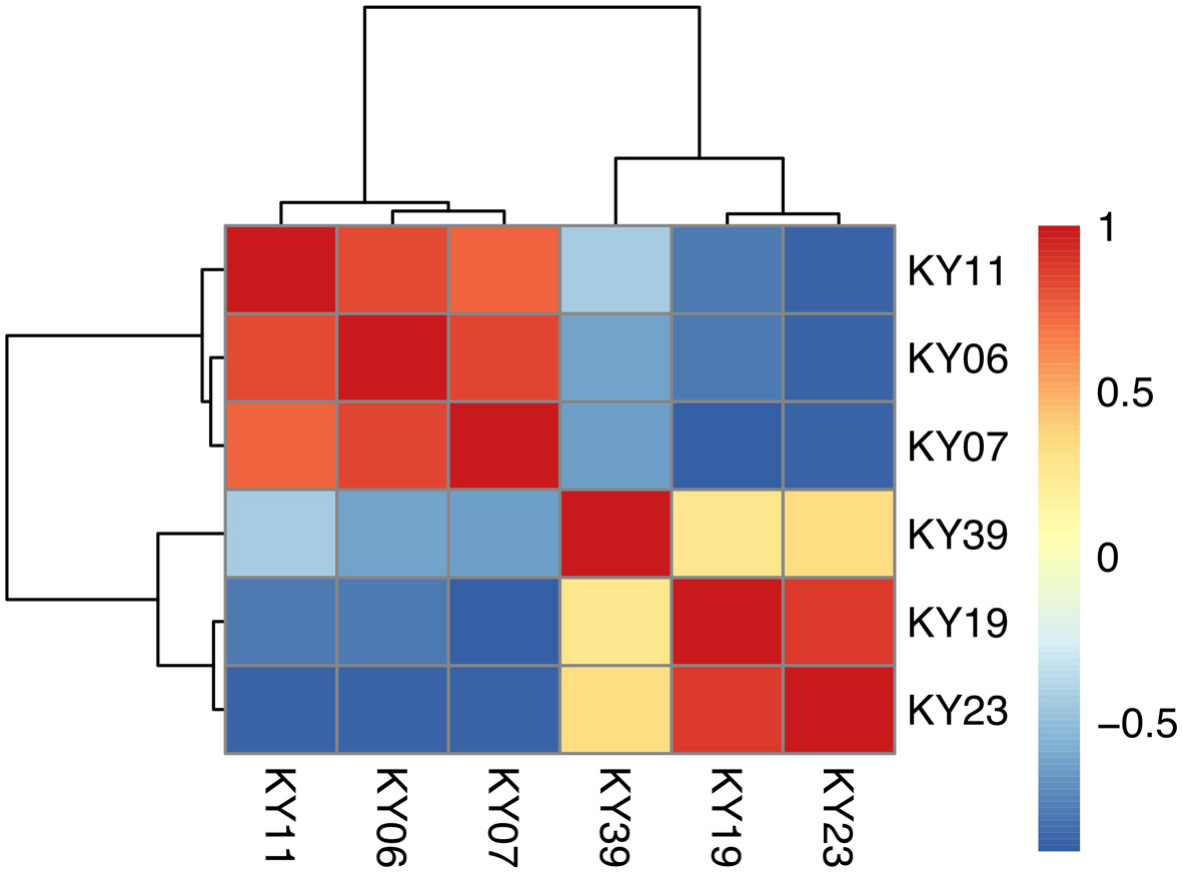
Hierarchical clustering of *Pd* gene expression on bats with WNS. Hierarchical clustering of differentially expressed Pd genes using Pearson correlation complete-linkage clustering. Scale shows Pearson correlation coefficient.

To examine the gene expression of the *Pd* pathogen using a dual RNA-Seq approach [72], we separately generated a genome-guided Trinity assembly (S3 Dataset) with the Broad Institute *G*. *destructans* genome. The reads from each of the WNS-affected tissues were mapped onto this assembly with Bowtie and gene expression estimated using RSEM (S4 Dataset). Expression levels for the *Pd* genes with the greatest variance are shown in Fig 5. Hierarchical clustering (Fig 6) and principal component analysis (S2 Fig) of the differentially expressed transcripts indicated that *Pd* gene expression was most similar in the wing tissues from bats obtained from the same hibernaculum (Table 1). The expression patterns of *Pd* genes were more similar for KY06, KY07, and KY11, which corresponds to bats captured in Cave 1 in Kentucky, and for KY19 and KY23, which were captured from Cave 2.

The possible functions of the *Pd* genes expressed among the WNS-affected samples were analyzed by sequence homology. We first examined the expression levels of a family of secreted proteases that have been proposed to be involved in Pd virulence [73, 74] and found that these alkaline proteases were expressed by *Pd* in all 6 wing samples (Table 4). Destructin-2 was the most highly expressed isoform in all WNS-affected bat *Pd* samples.

**Table 4:** Expression of destructin transcripts in *Pd* growing on WNS-affected bats.

Isoform	UniProt Match	Protein Name	Putative Function(s)	KY06^1^	KY07	KY11	KY19	KY23	KY39
GG4320| c0_g1_i1	ALP_ACRCH	Destructin-2 (Alkaline protease-2)	Subtilisin-family alkaline protease	1004	711.7	1038	3696	4437	1157
GG6167| c0_g1_i1	ORYZ_ASPCL	Destructin-1 (Alkaline protease-1)	Subtilisin-family alkaline protease	516.8	107.4	720.0	779.4	192.4	128.5
GG5612| c0_g1_i1	ORYZ_NEOFI	Destructin-3	Subtilisin-family alkaline protease	8.3	19.2	23.6	231.1	26.2	12.9

^1^ Trimmed mean of M-values- (TMM-) normalized expression determined by RSEM in fragments per kilobase of transcript per million mapped reads (FPKM).

We next examined the *Pd* transcript clusters for additional factors that could affect virulence. Alignment by BLAST to the Swissprot and Uniprot90 databases identified 12 056 transcripts with significant homology to known fungal genes (S8 Table). For the remaining 67 *Pd* transcript clusters, Trinotate was not able to identify known functional domains or signal peptides present in these previously uncharacterized *Pd* transcripts. The results from the BLAST alignment were examined for genes known to be involved in processes that could affect *Pd* virulence, such as secreted proteases [73–75], metal binding proteins [76], fungal cell wall remodeling [76, 77], and other virulence factors [75, 77, 78]. This analysis identified 46 *Pd* genes that could be involved in pathogenesis (Table 5), including additional secreted proteases that could be involved in tissue invasion.

**Table 5:** Possible virulence genes expressed in *Pd* growing on WNS-affected bats.

*Pd* Gene	UniProt Match	Protein Name	Putative Function	TMM-normalized expression by RSEM (FPKM)					
				KY06	KY07	KY11	KY19	KY23	KY39
GG1839|c0_g1	MSBP2_ARATH	Membrane steroid-binding protein 2	Antimicrobial peptide response	63.6	146.4	30.1	24.8	65.3	48.2
GG5000|c0_g1	DUR3_YEAST	Urea active transporter	Antimicrobial peptide susceptibility	65.7	79.5	107.0	51.6	19.8	14.6
GG5000|c1_g1	DUR31_SCHPO	Probable urea active transporter 1	Antimicrobial peptide susceptibility	0	79.5	0	81.1	102.1	0
GG680|c0_g1	GMDG_00178	Uncharacterized protein	Drug resistance transporter	0	29.7	24.4	141.0	175.2	39.8
GG2580|c0_g1	SOL5_ALTSO	Bifunctional solanapyrone synthase	Phytotoxin synthesis, Pathogenesis	101.4	0	0	157.3	99.2	0
GG6092|c0_g1	SOL5_ALTSO	Bifunctional solanapyrone synthase	Phytotoxin synthesis, Pathogenesis	66.4	35.8	113.6	714.7	1116	352.5
GG3668|c0_g1	TOXA_COCCA	Putative HC-toxin efflux carrier TOXA	Toxin transporter	20.4	0	77.5	143.8	85.0	62.7
GG3668|c1_g1	TOXA_COCCA	Putative HC-toxin efflux carrier TOXA	Toxin transporter	78.6	0	74.4	122.5	40.1	0
GG432|c0_g1	TOXA_COCCA	Putative HC-toxin efflux carrier TOXA	Toxin transporter	114.0	35.1	54.2	89.5	131.9	53.1
GG432|c1_g1	TOXA_COCCA	Putative HC-toxin efflux carrier TOXA	Toxin transporter	42.5	49.0	20.2	66.5	132.5	0
GG680|c1_g1	TOXA_COCCA	Putative HC-toxin efflux carrier TOXA	Toxin transporter	27.1	20.9	12.9	148.7	198.9	0
GG1568|c0_g1	CHI1_COCP7	Endochitinase 1	Fungal cell wall	305.2	26.9	348.1	581.9	46.0	27.2
GG2156|c0_g1	YCZ2_SCHPO	Putative mannan endo-1,6-alpha-mannosidase	Fungal cell wall	32.5	6.3	7.7	57.4	214.3	113.9
GG1843|c0_g1	ENG1_SCHPO	Endo-1,3(4)-beta-glucanase 1	Fungal cell wall	77.7	0	62.1	196.4	8.6	26.0
GG2744|c0_g1	BGLE_NEOFI	Probable beta-glucosidase E	Fungal cell wall	115.2	355.1	84.6	65.8	77.6	8.2
GG2744|c1_g1	BGLE_NEOFI	Probable beta-glucosidase E	Fungal cell wall	0	238.0	0	0	124.6	0
GG4455|c0_g1	AVR4_PASFU	Race-specific elicitor A4	Fungal cell wall, Pathogenesis	106.3	5.1	158.0	866.6	309.2	166.0
GG2938|c0_g1	BGBP_PENMO	Beta-1,3-glucan-binding protein	Innate immune activation	121.2	85.2	150.4	34.5	68.4	104.5
GG2938|c0_g2	BGBP_PENMO	Beta-1,3-glucan-binding protein	Innate immune activation	840.1	332.2	768.8	783.6	476.6	339.3
GG6498|c0_g1	SR1A_PHYPO	Spherulin-1A	Manganese binding	1067	629.2	1202	217.1	373.4	6328
GG3236|c0_g1	SBNA_RALME	Pd uncharacterized protein L8FZ85	Siderophore biosynthesis	68.3	205.4	60.5	48.3	192.0	0
GG4315|c0_g2	FRE3_YEAST	Ferric reductase transmembrane component 3	Siderophore transport, Iron binding	157.9	86.4	135.7	76.1	86.4	95.6
GG5300|c0_g1	FRE3_YEAST	Ferric reductase transmembrane component 3	Siderophore transport, Iron binding	2422	1441	1623	510.9	400.3	197.0
GG6235|c0_g1	SIT1_YEAST	Siderophore iron transporter 1	Siderophore transporter	0	0	0	23.1	135.3	0
GG6235|c0_g2	SIT1_YEAST	Siderophore iron transporter 2	Siderophore transporter	115.7	133.8	170.1	191.2	74.4	107.0
GG2118|c0_g2	ZRT1_YEAST	Zinc-regulated transporter 1	Zinc transporter	1873	1637	1447	924.5	1211	501.3
GG5016|c0_g2	ZRT1_YEAST	Zinc-regulated transporter 2	Zinc transporter	457.4	310.7	449.3	66.9	137.0	564.1
GG3064|c0_g1	MIRB_EMENI	Siderophore iron transporter mirB	Response to iron ion starvation	547.5	169.3	220.7	136.1	425.8	259.6
GG861|c0_g1	MIRB_EMENI	Siderophore iron transporter mirB	Response to iron ion starvation	656.0	410.1	712.3	913.4	1324	541.4
GG4694|c1_g1	LAC2_PODAS	Laccase-2	Melanin biosynthesis	64.2	19.8	329.4	553.8	69.1	179.0
GG6611|c0_g1	UREA_ASPFU	Urease	Nitrogen metabolism	49.8	0	0	38.8	151.1	222.7
GG6611|c1_g1	UREA_ASPFU	Urease	Nitrogen metabolism	0	28.2	34.8	28.6	112.6	0
GG6611|c3_g1	UREA_ASPFU	Urease	Nitrogen metabolism	0	54.8	0	55.7	142.6	0
GG6612|c0_g1	UREA_ASPFU	Urease	Nitrogen metabolism	0	0	0	499.6	0	0
GG3518|c0_g1	ALL2_ASPFU	Major allergen Asp f 2	Metallopeptidase, Fungal allergen	2789	4105	1021	3510	5373	449.6
GG2311|c0_g1	PRTA_ASPNG	Aspergillopepsin-2	Aspartic endopeptidase	59.3	79.9	84.5	709.2	1149	68.8
GG2082|c0_g1	PEPA_ASPOR	Aspartic protease pep1	Secreted aspartic endopeptidase	64.5	31.0	45.9	95.5	370.1	0
GG2082|c0_g2	PEPA_ASPOR	Aspartic protease pep1	Secreted aspartic endopeptidase	0.1	0.1	0.1	189.7	5.1	0
GG4492|c0_g1	CARP_CRYPA	Endothiapepsin	Secreted aspartic endopeptidase	648.7	460.5	311.8	406.5	423.6	216.0
GG448|c0_g1	LAP1_SCLS1	Leucine aminopeptidase 1	Secreted leucyl endopeptidase	100.7	84.0	95.7	283.3	265.6	183.0
GG788|c0_g1	SPM1_MAGO7	Subtilisin-like proteinase Spm1	Secreted serine endopeptidase	956.8	952.7	525.5	1260	1176	919.4
GG2765|c0_g1	PEPS_ASPPH	Carboxypeptidase cpdS	Serine carboxypeptidase	83.2	114.5	146.9	775.2	981.3	408.6
GG3562|c1_g1	SED4_ARTOC	Tripeptidyl-peptidase SED4	Serine endopeptidase, Pathogenesis	715.7	366.5	690.3	1703	2009	191.9
GG2259|c0_g1	SOD6_CANAL	Cell surface superoxide dismutase [Cu-Zn] 6	Superoxide metabolism, Pathogenesis	43.5	44.6	13.8	556.7	880.6	179.3
GG4408|c0_g1	CCPR2_ASPFU	Putative heme-binding peroxidase	Oxidative stress response, Iron binding	1044	842.8	477.1	63.0	42.3	421.4
GG6788|c0_g1	HOG1_CRYPA	Mitogen-activated protein kinase HOG1	Virulence and conidia formation	735.1	816.2	959.1	325.7	373.5	772.8

Because the tissue samples were collected from bats from 6 different hibernacula for this study, it is possible that differences in host or pathogen gene expression reflect differences in the environmental conditions present in each location, including the microbiome. In addition, the housing of the unaffected bats in captivity for 13 weeks prior to analysis could also have affected the microbiome. To examine the differences in the skin microbiome between the bats, we used MG-RAST to identify the lowest common ancestor of metagenomic sequences present (S8 Table). Although there were some differences observed in the bacterial microbiomes present on the wings of the 11 bats, there were no significant changes between the WNS-affected and unaffected samples when bacteria were identified at the class level. Several strains of *Pseudomonas fluorescens* isolated from bat tissues have been identified with *Pd* growth inhibiting properties [79]. MG-RAST analysis showed that *Pseudomonas* species are present in all 11 samples (S8 Table). *P*. *fluorescens* transcripts represented 2.8±0.6% of transcripts identified from gammaproteobacteria and 0.40±0.05% of all bacteria on the wings of unaffected bats and 0.37±0.07% of all bacteria on WNS-affected bats. *P*. *fluorescens* was present on all little brown myotis sampled, but was rare and relative abundance was not statistically different between WNS-affected and unaffected bats (p = 0.49, t = -0.71, df = 9).

## Discussion

The comparison of host gene expression between WNS-affected and unaffected little brown myotis clearly demonstrates that *Pd* infection causes physiological responses in wing tissue, where substantial fungal invasion of the skin occurs in WNS-affected bats [8]. The changes in transcript levels that we have observed indicate that host responses to fungal infection remain intact during hibernation and are similar to those observed during the initial stages of fungal infection in euthermic mammals [32]. These host responses include acute inflammation, wound healing, and metabolic changes. Pathogen gene expression varies among bats with WNS, suggesting host-pathogen interactions that mediate pathogenesis. Together, these results lay a foundation to determine which host and pathogen responses contribute to WNS resistance and susceptibility and identify targets to increase survival.

### Host Response to *Pd* Infection

The gene expression changes we observed in the wing tissue of WNS-affected bats are similar to those observed in other cutaneous fungal infections [80]. Cutaneous *Candida albicans* infections in humans and mice typically initiate an immune response by activating pattern recognition receptors of the C-type lectin family [81–83] and the toll-like receptor family, both of which we found upregulated in WNS-affected bat wing tissue (S4 Table). These included C-type lectin domain (CLEC) family members CLEC4D (MCL), CLEC4E (MINCLE), CLEC7A (Dectin-1), CLEC6A (Dectin-2), and Toll-like receptor 9. In mice and humans, protective host responses to *C*. *albicans* are usually characterized by many of the same cytokines and chemokines [29] that we have found upgregulated in WNS-affected wing tissue, including the cytokines IL-1β, IL-6, G-CSF, IL-23A, and IL-17C. Little brown myotis infected with *Pd* are increasing transcription of the key genes necessary for initiating a host response that provides protection from fungal infection. This clearly demonstrates that hibernation does not prevent innate immune responses in bats infected with *Pd* and that, although they are not closely related to rodents and primates [41], bats respond to fungal infections similarly to these other mammals.

The responses to *Pd* infection within bat wing tissue may be mediated by keratinocytes in the epithelial tissue. Activation of pattern recognition receptors by fungal ligands is expected to induce keratinocytes to produce many of the cytokines that we have found upregulated at the transcript level in WNS-affected bat wing tissue [84]. In addition to the cytokines typically involved in *C*. *albicans* responses described above, keratinocytes are also known to express the chemokine Ccl2 and the cytokines IL-20 and IL-24 in response to pattern recognition receptor activation [85]. Keratinocytes and fibroblasts are also known to exhibit a paracrine loop of IL-1 and IL-6 activation [86] that enhances wound healing and host defense to microbial infection and we found evidence of IL-1 and IL-6 receptor activation in the increased RNA levels for transcription factor p65, NFκB, and P-selectin glycoprotein ligand 1 (S4 Table). Another important cytokine produced by epithelial cells in response to infection is IL-17C [87]. This is an atypical IL-17 family member that is expressed by epithelial cells and causes autocrine responses in the epithelial cells that also express the IL-17RA and IL-17RE heterodimeric IL-17 receptor [87]. The wing tissue transcriptomes from WNS-affected and unaffected bats show similar expression levels of both IL-17RA and IL-17RE (S2 Dataset) and would, therefore, be expected to be responsive to IL-17C. The gene ontology analysis also found evidence for functional enrichment of genes involved in keratinocyte differentiation, presumably due to wound healing responses. Keratinocytes or other epithelial cells in bat wing tissue appear to have responded to the invasion of the epidermis by fungal hyphae.

Genes for pro-inflammatory mediators characterized the innate immune response that we observed in the wing tissue of *Pd* infected bats. Under euthermic conditions this would be expected to provide protection by the recruitment of monocytes and neutrophils, mediated by G-CSF, IL-23A, Ccl2, IL-17C and IL-6 [88], and the initiation of an adaptive Th17 or Th1 response. However, under the constraints of hibernation, responses that require leukocyte migration do not appear to occur in *Pd*-infected bats. We do not find strong evidence of increased expression for genes characteristic of either innate or adaptive leukocytes, except for L-selectin, which is expressed on T cells, and CD177, which is expressed on neutrophils. Lower than expected levels of monocyte, neutrophil, Th1, and Th17 cell recruitment may be related to the sequestration of leukocytes during hibernation [45]. However, we have observed neutrophil recruitment in hibernating little brown myotis in response to another fungal infection (Table 1). In the histological examination of the current samples, we found neutrophilic inflammation in both WNS-affected and unaffected wing tissue (Table 1). However, this inflammation did not occur at the sites of *Pd* infection. Curiously, we found a significant increase in WNS-affected tissue for transcripts for CD3γ and CD45 that could be expressed by gamma-delta T cells or other innate lymphocytes that reside in the skin [89]. It is possible that *Pd* is specifically suppressing neutrophil and/or T cell recruitment by interfering with chemotactic signals, similar to the suppression of inflammatory immune responses during chytridiomycosis in amphibians [42]. However, analysis of tissue levels of the cytokines and chemokines is necessary to confirm the secretion of these proteins. Because neutrophils and T cells do not appear to be recruited to sites of *Pd* infection during hibernation, only local inflammatory mediators may be available and they appear to be unable to control the infection in little brown myotis.

In addition to immune responses, hibernating bats also respond to *Pd* infection in other ways. We found transcripts for proteins from many pathways involved in metabolism, signaling, gene expression, transport, migration, and differentiation that were altered in WNS-affected bats (Fig 4). We cannot exclude the possibility that some of these differences were due to the different hibernation conditions of the two groups of bats. However, the differential expression of the genes in these pathways demonstrates that they are subject to regulation during hibernation and can respond to infection, tissue damage, and/or environmental changes.

Host responses to fungal infection can be influenced by changes in the pathogen, including gene expression changes in the colonizing fungus, such as *C*. *albicans* [29]. We found significant variability in the gene expression by *Pd*, which is particularly interesting because all *Pd* in North America is presumed to be a clone of the same mating type [90]. The pathogen has adopted different gene expression profiles in the 6 bat tissues (Fig 5), perhaps in response to differences in the host environments. Correspondingly, host gene expression patterns also show differences between the WNS-affected tissue samples. Of particular interest is the observation that the cytokine and chemokine genes found in principal component 2 of our PCA analysis (Fig 3B and S5 Table) are expressed at very different levels in the 6 *Pd*-infected samples. From this study we cannot determine whether the differences in pathogen gene expression are driven by differences in the host environment or vice versa. Although all 6 WNS-affected bats had visible signs of WNS, had similar *Pd* burdens, and similar histopathology, it is possible that the differences in host or pathogen gene expression that we observed may have affected progression of WNS and survival.

### Responses that May Contribute to WNS Mortality

Because the increased frequency of arousals from torpor appears to be a primary cause of WNS mortality [5, 14, 22], we considered possible mechanisms that could affect torpor bout length. The increased gene expression of IL-1, IL-6, and other pro-inflammatory cytokines mediates a local acute inflammatory response to *Pd*. These cytokines also have systemic effects that modify behavior and thermoregulation [91]. In addition to cytokine and chemokine transcript increases, we also found increased transcripts for the enzyme cyclooxygenase-2 (prostaglandin G/H synthase 2) and both secreted and cytosolic phospholipase A2 that form critical inflammatory lipid mediators such as prostaglandin H_2_. The eicosanoids generated by these enzymes, along with the actions of the upregulated genes kallikrein-6 and cathepsin S, are expected to generate pain and itching by locally activating neuronal nociceptors [92, 93]. This, in turn, could affect torpor bout length and/or behavior during periodic arousals. Indeed, we have documented significantly more grooming in WNS-affected bats infected in the wild [94], although a different study on laboratory-infected bats did not find similar behavior changes [95]. Together, the upregulated genes will likely generate an inflammatory microenvironment within the wing that may contribute to the robust wound healing response that we observe in WNS-affected bats. However, inflammation can also play a detrimental role in some diseases [96]. Further tissue damage and subsequent wound healing occurs in surviving bats upon emergence from hibernation [19]. These local affects of inflammation (pain and itching) as well as systemic effects are likely to play a key role in WNS pathology.

In addition to the gene expression changes that may contribute to acute inflammation locally within the epithelial tissues invaded by *Pd*, the systemic release of febrile cytokines such as IL-6 could affect the signals that control hibernation arousal. However, an exogenous pyrogen, lipopolysaccharide, is not able to provoke arousals in hibernating golden-mantled ground squirrels [97], so it may be unlikely that inflammation or febrile cytokines can directly trigger arousal in WNS-affected bats. Intracerebroventricular injection of prostaglandin E_2_ in golden-mantled ground squirrels induces arousal from torpor and a febrile response during an extended periodic arousal [97]. Our observation of increased expression of the enzyme that generates prostaglandin H_2_ may provide a mechanism that explains the shortened torpor bouts in WNS-affected bats, if it can be shown that this enzyme is active in the tissue and produces enough prostaglandin H_2_ to act systemically.

In addition to the changes in expression of genes involved in immune responses and wound healing, we also found significant changes in metabolic genes. We found evidence of gene expression changes consistent with increased fat metabolism, including changes in transcripts for apolipoproteins, lipid transport proteins, protein metabolism, and carbohydrate metabolism. Of particular interest, we found increases in the expression of hydroxycarboxylic acid receptors 2 and 3 that are known to mediate adiponectin secretion [98]. This suggests that infection with *Pd* may directly trigger changes in lipid and carbohydrate metabolism that contribute to WNS pathology. These changes stand in contrast to the changes that have been seen in the brain transcriptome of hibernating horseshoe bats (*Rhinolophus ferrumequinum*) [99] and the brain proteome of hibernating Rickett’s big-footed bats (*Myotis pilosus*) [100], which show decreased fat metabolism during torpor. By leading to premature depletion of fat stores, these gene expression changes could contribute to WNS mortality.

The other changes in host gene expression that we observed are consistent with a multi-stage progression model of WNS [22]. We also found support for changes in genes involved in oxidative stress [23] and body fluid levels, which may contribute to WNS progression. Together, the pattern of gene expression changes that we find in little brown myotis with WNS suggests that a combination of maladaptive responses may contribute to mortality. However, the number of upregulated genes involved in the acute inflammatory response suggests that excessive inflammation may also be a factor contributing to pathology even prior to emergence from hibernation when it is suspected to contribute to wing damage [101].

### Implications for Future Studies

The changes in host transcript levels that we have found are presumably caused by physiological responses of the host to infection. However, caution must be used when extending these transcriptional responses to functional mechanisms because the current study does not measure protein or metabolite levels directly. Future studies will be necessary to determine which of the gene expression changes observed affect which host response mechanisms.

The little brown myotis chosen for the WNS-affected samples were exhibiting WNS pathology and appeared unlikely to survive at the time of sample collection. For this reason, it is presently uncertain which of the gene expression changes that we have observed are contributing to protection and which are pathological. Another factor that likely contributes to the variation in gene expression that we observed among the samples collected from free-ranging bats is the time since the most recent arousal from torpor. Prior to collection of each wing tissue sample, bats were artificially aroused for 30 to 120 minutes. This period of arousal is similar in duration to the natural arousals during hibernation for little brown myotis [14], and presumably of sufficient duration for some innate immune responses to occur and for transcript levels to be altered. One reason for this procedure was to avoid disparities between the elapsed time from the most recent arousal bout until tissue collection. For the WNS-affected bats we could not determine when the most recent natural arousal would have occurred, but it would have likely been more recently than in unaffected animals, as affected animals arouse from torpor more frequently [14]. In the current study we cannot resolve whether the changes in gene expression that we observed occurred during the most recent arousal, during previous periodic arousals, or during torpor. Future studies will be needed to determine which of the changes in gene expression that we observed during WNS in bats in the wild also vary in controlled captive hibernation conditions when prior arousal patterns are known. Further studies are also needed to compare the physiological responses in bats exhibiting WNS morbidity to responses in less susceptible bats, such as European species, North American species that are less susceptible like the big brown bat [11], and the remnant populations of little brown myotis that appear to have developed tolerance or resistance to *Pd* [1]. Such studies should point to a path forward for bats in North America to persist in a landscape where *Pd* is endemic.

### Conclusions

Little brown myotis mount a host response to *Pd* infection during hibernation. Which components of this response are protective or contribute to WNS pathology remains to be resolved. The innate immune response we have observed would be expected to promote a Th17-directed adaptive immune response that could clear the infection. However, the energetic constraints of hibernation may prevent little brown myotis from execution of the Th17- and neutrophil-mediated phases of the immune response. This may lead to excessive inflammatory responses, either during hibernation or upon emergence. The changes in host gene expression that we observed demonstrate that during *Pd* infection, little brown myotis also alter other defense responses, metabolic pathways, and transcription. Numerous *Pd* genes that may contribute to virulence were identified and these represent potential pathogen responses to host defense. Hibernation does not prevent a host response to infection and a better understanding of the differences between host and pathogen responses in bats susceptible to WNS and those resistant may lead to ways for increasing survival.

## Materials and Methods

### Ethics Statement

This study was carried out on bats from non-endangered species in strict accordance with the recommendations in the Guide for the Care and Use of Laboratory Animals of the National Institutes of Health. All methods were approved by the Institutional Animal Care and Use Committee at Bucknell University (protocol DMR-016). Animals were humanely euthanized by isoflurane anesthesia overdose followed by decapitation. In Illinois, animal collection was conducted by state wildlife officials and a numbered permit was not required. Scientific collector’s permits were obtained in Michigan (SC1448), Minnesota (201174), and Kentucky (SC1411147).

### Samples

We collected hibernating little brown myotis from cave or mine walls at the locations listed in Table 1. Bats collected from all locations are expected to be from the same genetic population of eastern little brown myotis [102]. For bats unaffected by WNS, little brown myotis were first swabbed on the left forearm for quantitative PCR analysis. After measurements were taken, bats were individually placed in cloth bags and hung in constant temperature thermoelectric coolers (Koolatron PC-3) maintained at ~7°C. Water-saturated sponges were placed in the bottom of each cooler to maintain humidity during transportation to Bucknell University. Bats were housed for 13 weeks in a Percival (model I36VLC8) environmental chamber with conditions set to 4°C and 95% relative humidity. Bats were provided water throughout hibernation. Bats were aroused from hibernation for 30–120 minutes prior to euthanasia. For WNS-affected bats, little brown myotis were collected in the field, measured, swabbed for quantitative PCR, and humanely euthanized after being aroused from hibernation for 60–120 minutes. Scaled mass index (SMI) was calculated using the formula (mass(in g))*(38.01/(forearm length(in mm))^1.406 [103]. Wing tissue was placed in formalin for histology and placed in RNAlater (Sigma-Aldrich) for gene expression analysis. RNAlater samples were stored at ambient temperature for up to 24 hours before long-term storage at -80°C. RNA was purified from 50 mg of wing tissue using a QIAGEN RNeasy Mini Kit. All samples used for RNA sequencing had RNA integrity values greater than 7.0 using an Agilent Bioanalyzer.

### Verification of WNS Status

Wing skin tissue was removed from the bones of the arm and digits and rolled onto 2 cm paraffin wax logs. The logs were then fixed in 10% neutral buffered formalin for at least 24 hours. Each log was cut into 3 pieces that were processed into paraffin blocks overnight in a Tissue-Tek VIP processor (Sakura Finetek). The pieces were embedded in paraffin blocks, sectioned at 3 microns, and stained with periodic acid Schiff with a hematoxylin counterstain [8]. WNS lesions (Table 1; WNS) were identified as cupping erosions with fungal hyphae and conida present. Inflammatory foci (Table 1; Infl) were identified as clusters of infiltrating neutrophils and were not associated with the asymmetrical curved conidia of *Pd*.

To determine presence or absence of *Pd* on bats unaffected by WNS, each swab was tested twice by quantitative PCR [64] by Jeffrey T. Foster at University of New Hampshire. A cycle-threshold less than 40 was used as a positive result. One of the 5 unaffected bats had one positive and one negative test (Table 1), but histology (Table 1) and subsequent RNA sequencing determined this to most likely be a false positive (S2 Table; p = 2.2x10^-6^). For bats affected by WNS, we performed quantitative PCR to measure the *Pd* load, in genomic equivalents normalized to swabs spiked with 10 000 *Pd* conidia, that were detected on each bat [15].

### Next Generation RNA Sequencing

The Genome Sequencing and Analysis Facility at the University of Texas at Austin performed all library preparation and quality control procedures. Directional RNA libraries were prepared with poly-A mRNA enrichment, dUTP/UDG strand-specific labeling, fragmentation, and 200 base pair size selection. RNA-Seq was performed in two lanes of an Illumina HiSeq 2500 with 101 base pair length reads obtained.

### Transcriptome Assemblies

The paired reads from all samples were preprocessed by removing adapters and using trimmomatic PE [104] with settings of Illumina clip:2:30:10, seed mismatches:2, palindrome threshold:30, clip threshold:10, leading:5, trailing:5, minlength:36. The remaining paired reads were then combined and Trinity (v2.0.4) was used in strand-specific mode (RF) to construct a de novo assembly [105]. K-mer in silico read normalization with maximum coverage of 50 resulted in 22 482 456 read pairs that were used for assembly out of 177 755 004 total. The assembly was then filtered to remove *Pd* sequences using the program Deconseq [65] with the Broad Institute *Geomyces destructans* genome 20631–21 used to identify pathogen sequences and with the little brown myotis genome (Myoluc2.0) used to retain host sequences. Bowtie 1.0.1 [106] was used to determine the number of reads that mapped to each transcript in the assembly.

### Differential Expression

The script align_and_estimate_abundance.pl included in the Trinity v2.0.6 distribution [105] was used to estimate expression levels for each transcript. Bowtie 1.0.1 [106] was used to map reads (including unpaired reads after quality trimming) from each sample onto the assembly. RSEM v1.2.20 [107] was used to apply an expectation maximization algorithm to predict gene expression counts for each transcript. Expression levels are presented after trimmed mean of M-values (TMM) normalization in fragments per kilobase of transcript per million mapped reads (FPKM). DESeq2 v1.8.1 [67] was used to determine the probability of differential expression for each Trinity transcript cluster that had a minimum RSEM-estimated count, before normalization, of 5 across all samples. For DESeq2 analysis, the default values for removing outliers and filtering lowly expressed transcripts were used. An alpha value of 0.05 was used instead of the default of 0.1 to decrease the number of differentially expressed genes identified. Posterior probabilities of differential expression for individual transcript isoforms were estimated using a Bayesian approach with EBSeq v1.8.0 [68]. False discovery rate [108] was used to control for multiple comparisons. NCBI BLAST v2.2.29+ [109] was used to identify the highest-ranking match for each isoform in the UniProt Swissprot database (downloaded on Sep 17, 2014) with an e-value cutoff of 1x10^-5^.

Hierarchical clustering of samples and genes was performed within R 3.1.2 using the hclust function with the complete linkage method. Bootstrap analysis of clustering was performed using the pvclust 1.3–2 package and 1000 replications [69]. Principal component analysis was performed using the prcomp function and visualized with the rgl 0.93.1098 package.

### Gene Ontology

NCBI BLAST v2.2.29+ [109] was used with an e-value cutoff of 1x10^-5^ to identify homologs in the Uniprot Swissprot human protein database (downloaded on Nov 25, 2014) for transcripts significantly upregulated in WNS-affected bat wing tissue with an FDR of less than 0.1 (in order to increase the number of genes prior to subsequent analysis with higher stringency FDR). Unique Ensembl gene IDs were identified for 1144 of the 1922 upregulated transcripts and 481 of the 1356 downregulated transcripts. GOrilla [70] was used with a p value cutoff of 0.001 to identify upregulated or downregulated biological processes by comparison to the background list of 12 828 human genes identified by BLAST in the Trinity assembly. Multiple testing correction [108] was used with an FDR cutoff of 0.01. Results were visualized as a treemap with REVIGO [71].

### *Pd* Gene Analysis

Trinity v2.0.4 was used to generate a *Pd* assembly in genome-guided mode with jaccard clipping and using the Broad Institute *G*. *destructans* genome 20631–21. This assembly was used to assess pathogen gene expression in the samples from WNS-affected bats using RSEM v1.2.20 [107]. Trinotate v2 was used to annotate the *Pd* transcripts by using NCBI BLAST v2.2.29+ [109] and both the Swissprot and Uniref90 databases (downloaded on Sep 17, 2014).

### Metagenome Analysis

Reads for each sample were analyzed using MG-RAST v.3.5 [110] to identify metagenomic sequences after filtering against the *B*. *taurus* genome (the taxonomically closest genome available for filtering). For assignment of organism abundance, the best hit classification was used with the M5NR database, maximum e-value cutoff of 1x10^-5^, minimum identity cutoff of 60%, and minimum alignment length cutoff of 15.

## Supporting Information

S1 Fig MA plot of gene expression using the trinity transcriptome assembly. Expression levels for every gene are shown by comparing RSEM-estimated counts to the fold-change in expression between unaffected and WNS-affected bat tissues. Blue points indicate significant differential expression determined by DESeq2 using an FDR cutoff of 0.05. Genes that are more highly expressed in WNS-affected tissues are found in the lower side of the graph.
(TIF)

S2 Fig Principal component analysis of *Pd* genes. The Trinity utility PtR was used to conduct principal component analysis on the *Pd* genes with a minimum expression of 10 FPKM.
(PDF)

S1 Table Read statistics of RNA-Seq samples. (DOCX)

S2 Table FPKM analysis of *Pd*-derived transcripts prior to removal. (DOCX)

S3 Table Transcriptome assembly comparison. (DOCX)

S4 Table Differentially expressed genes determined by RSEM and DESeq2 combined with EBSeq and trinotate results. (XLSX)

S5 Table Principal component analysis rotation values. (XLSX)

S6 Table Differentially expressed genes used for GOrilla analysis. (XLSX)

S7 Table Gene ontology biological process categories over-represented in WNS-affected tissues. (XLSX)

S8 Table *Pd* gene expression estimated by RSEM combined with trinotate results. (XLSX)

S9 Table MG-RAST analysis of best hit classification for bacterial genes. (XLSX)

S1 Dataset FASTA file of de novo assembly of little brown myotis transcriptome. (ZIP)

S2 Dataset RSEM gene expression matrices used for differential host gene expression calculations. (ZIP)

S3 Dataset FASTA file of genome-guided trinity assembly of *Pd* transcriptome. (ZIP)

S4 Dataset RSEM gene expression matrices for *Pd* transcripts. (ZIP)
