# Supplementary figures and images for: The White-Nose Syndrome Transcriptome: Activation of Anti-fungal Host Responses in Wing Tissue of Hibernating Little Brown Myotis
Source: PLoS Pathog. 2015 Oct 1;11(10):e1005168. doi: 10.1371/journal.ppat.1005168 (PMC4591128; doi:10.1371/journal.ppat.1005168)

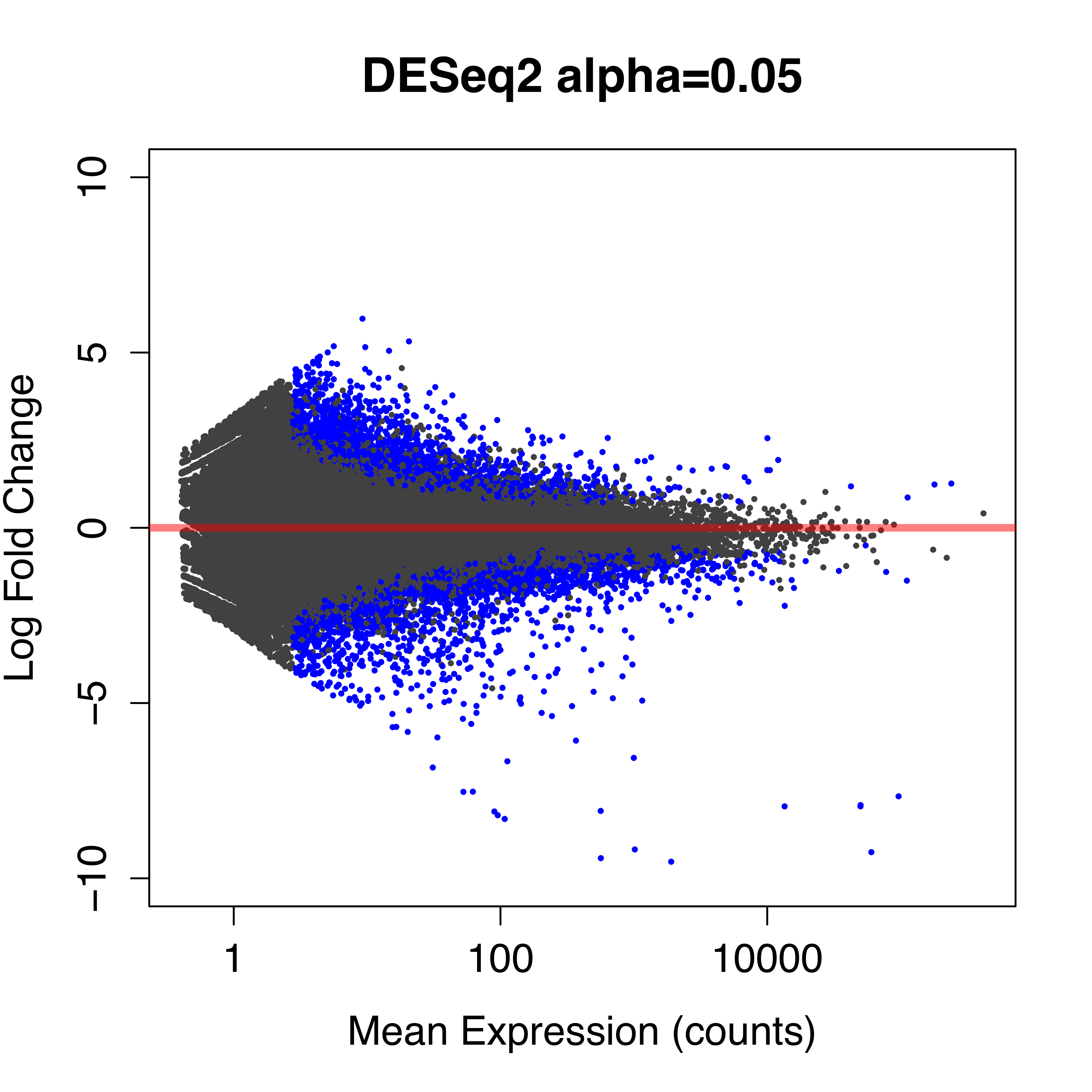

Supplement: S1 Fig — Expression levels for every gene are shown by comparing RSEM-estimated counts to the fold-change in expression between unaffected and WNS-affected bat tissues. Blue points indicate significant differential expression determined by DESeq2 using an FDR cutoff of 0.05. Genes that are more highly expressed in WNS-affected tissues are found in the lower side of the graph. (TIF) [file ppat.1005168.s001.tif]

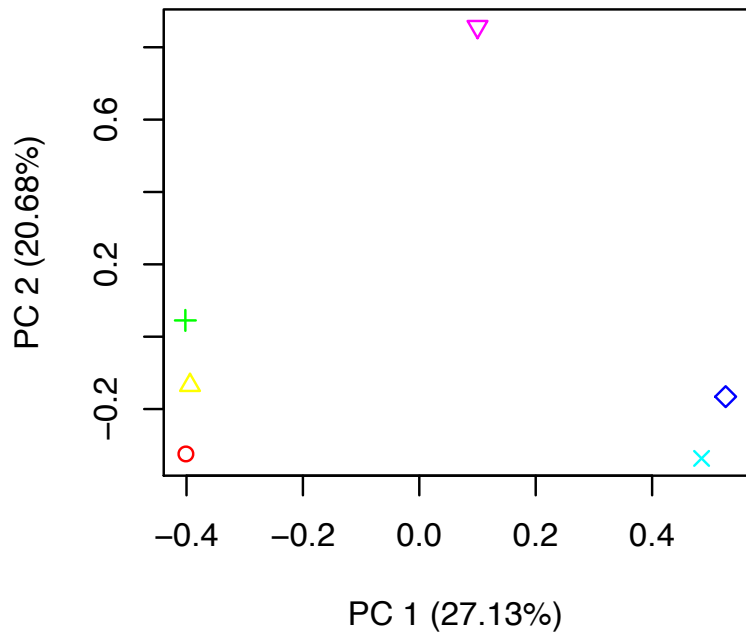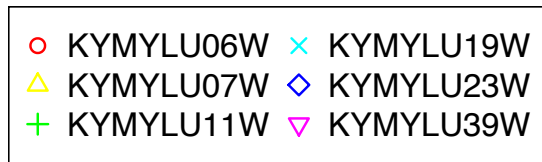

Supplement: S2 Fig — The Trinity utility PtR was used to conduct principal component analysis on the Pd genes with a minimum expression of 10 FPKM. (PDF) [file ppat.1005168.s002.pdf]
