# Supplementary material for: The White-Nose Syndrome Transcriptome: Activation of Anti-fungal Host Responses in Wing Tissue of Hibernating Little Brown Myotis
Source: PLoS Pathog. 2015 Oct 1;11(10):e1005168. doi: 10.1371/journal.ppat.1005168 (PMC4591128; doi:10.1371/journal.ppat.1005168)
Supplement: S1 Table — (DOCX) [file ppat.1005168.s003.docx]

**Table S1.** RNA-Seq Samples

| Sample | RIN^1^ | Insert Size^2^ | Initial Read Pairs | Paired Reads QC^3^ |
| --- | --- | --- | --- | --- |
| MI011 | 8.9 | 495 | 19 448 065 | 17 269 150 |
| MN064 | 8.8 | 461 | 17 306 228 | 15 610 861 |
| MN075 | 8.1 | 375 | 17 684 763 | 15 833 990 |
| MN090 | 8.2 | 382 | 17 468 515 | 15 549 958 |
| IL114 | 8.0 | 422 | 16 800 298 | 15 092 645 |
| KY06 | >7 | 468 | 19 451 528 | 17 647 644 |
| KY07 | 7.4 | 451 | 19 018 600 | 17 350 018 |
| KY11 | 7.7 | 482 | 19 463 717 | 17 733 760 |
| KY19 | 8.3 | 421 | 17 842 268 | 15 857 998 |
| KY23 | 7.9 | 375 | 15 186 880 | 13 528 720 |
| KY39 | >7 | 395 | 17 780 093 | 16 280 260 |

^1^ RNA Integrity Number determined by Agilent Bioanalyzer using a Total RNA Nano chip. “>7” indicates that the automated peak finding software did not accurately identify the 18S and 28S peaks, but that the electropherogram profile was comparable to other samples with RIN values greater than 7.

^2^ After library prep, Agilent Bioanalyzer was used to measure average insert size of cDNA using a high sensitivity chip.

^3^ After trimmomatic quality control, the number of properly paired reads remaining that were used for transcriptome assembly.
