# Supplementary material for: The White-Nose Syndrome Transcriptome: Activation of Anti-fungal Host Responses in Wing Tissue of Hibernating Little Brown Myotis
Source: PLoS Pathog. 2015 Oct 1;11(10):e1005168. doi: 10.1371/journal.ppat.1005168 (PMC4591128; doi:10.1371/journal.ppat.1005168)
Supplement: S2 Table — (DOCX) [file ppat.1005168.s004.docx]

| Sample | *Pd* genes expressed^1,2^ |
| --- | --- |
| MI011 | 74 |
| MN064 | 70 |
| MN075 | 406 |
| MN090 | 94 |
| IL114 | 84 |
| KY06 | 7931 |
| KY07 | 8554 |
| KY11 | 8409 |
| KY19 | 8891 |
| KY23 | 7525 |
| KY39 | 6468 |

^1^Number of genes expressed by at least 1 FPKM (fragments per kilobase of transcript per million mapped reads)

^2^Putative *Pd* genes were identified in the de novo Trinity assembly by deconseq. Subsequent mapping of RNA-Seq reads onto this subset was performed with bowtie and RSEM was used to estimate coverage. *Pd* genes apparently expressed in bats without *Pd* exposure presumably represent either non-*Pd* genes in the *Pd* assembly or mis-mapping of non-*Pd* RNA reads to *Pd* genes due to sequence similarity.
