# Supplementary material for: The White-Nose Syndrome Transcriptome: Activation of Anti-fungal Host Responses in Wing Tissue of Hibernating Little Brown Myotis
Source: PLoS Pathog. 2015 Oct 1;11(10):e1005168. doi: 10.1371/journal.ppat.1005168 (PMC4591128; doi:10.1371/journal.ppat.1005168)
Supplement: S3 Table — (DOCX) [file ppat.1005168.s005.docx]

**Table S3.** Transcriptome Assembly Statistics

|  | Trinity  de novo | Trinity  de novo -*Pd* | *Pd* Genome-guided Trinity |
| --- | --- | --- | --- |
| Genes | 254 381 | 238 749 | 11 605 |
| Transcripts | 363 349 | 346 499 | 11 838 |
| Contig N50^1^ | 1180 | 1205 | 708 |
| Median contig length | 394 | 392 | 426 |
| Inward-oriented pairs^2^ |  | 100% | 100% |
| Reads mapped^2^ |  | 87.2% | 1.0% |
| Insert size^2^ |  | 192.6±76.3 | 181.1±64.7 |
| Transcripts >80% full length by BLAST^3^ |  | 12 007 | 2312 |
| Transcripts >20% full length by BLAST^3^ |  | 78 721 | 5886 |
| Complete Single-copy BUSCOs^4^ | 653 (22%) | 660 (22%) | 458 (32%) |
| Complete Duplicated BUSCOs^4^ | 1497 (49%) | 1479 (49%) | 67 (5%) |
| Fragmented BUSCOs^4^ | 321 (11%) | 325 (11%) | 549 (38%) |
| Missing BUSCOs^4^ | 552 (18%) | 559 (18%) | 364 (25%) |
| Total BUSCO groups searched^4^ | 3023 | 3023 | 1438 |

^1^ N50 length is the length for which the collection of all contigs of that length or longer contains at least half of the sum of the lengths of all contigs, and for which the collection of all contigs of that length or shorter also contains at least half of the sum of the lengths of all contigs.

^2^ Samtools stats was used to determine the percentage of inward-oriented read pairs, the number of reads mapped, and the average insert size of paired reads after Bowtie alignment of the KY11 paired reads to each transcriptome assembly.

^3^ The best match for each transcript was determined by BLAST alignment against the Uniprot Swiss-prot database, except for the *Pd* assembly, which was mapped against the Uniref90 database.

^4^ Transcriptome assembly completeness as determined by Benchmarking Universal Single-Copy Orthologs using the vertebrate (first two assemblies) or fungi (third assembly) BUSCO profiles.
